# Creating the evidence for family medicine advocacy in Africa

**DOI:** 10.4102/phcfm.v17i1.5269

**Published:** 2025-11-18

**Authors:** Robert J. Mash

**Affiliations:** 1 Division of Family Medicine and Primary Care, Faculty of Medicine and Health Sciences, Stellenbosch University, Cape Town, South Africa

The World Organization of Family Doctors (WONCA) has members from only eight countries in Africa (South Africa, Botswana, Kenya, Uganda, Nigeria, Ghana, Liberia, and Zambia), although there are 48 countries listed in the region. The PRIMAFAMED (Primary Care and Family Medicine) network has had contact with around 25 countries, but many of them do not have a national association for family physicians, and the numbers are few. Does one conclude that family medicine is not that relevant in Africa or that African health systems are yet to realise the vital contribution that family physicians can make?

This begs the question – what evidence do we have that family physicians have an impact and are worth the investment? Globally, there is a wealth of evidence that family physicians (doctors with postgraduate training in family medicine or general practice) improve health outcomes and health services, but this is mostly from high-income countries with very different models of care.^1,2^

In Africa, there is evidence that family physicians make an impact, but much of the evidence is qualitative and observational, and comes from South Africa. This evidence tells us that healthcare workers and managers perceive family physicians to have an impact across six roles: as clinicians, consultants, capacity builders, clinical trainers, leaders of clinical governance and community-oriented primary care.^3^ They reported that family physicians had a greater impact in these roles when compared to medical officers without postgraduate training. District managers also perceived that family physicians improved the core functions of primary care and primary hospital care, particularly comprehensiveness and coordination, and the quality of clinical care through teaching, training and clinical governance activities.^4^

A special collection of short reports from across Africa suggested that family physicians have an impact on key inputs to the district health system, particularly the capacity of the workforce, supply of equipment, functioning of the health information system and use of digital technology.^5^ This series also described how family physicians improve the core functions of the health system (availability and utilisation of services, comprehensiveness, coordination, continuity and person-centredness) and clinical governance (quality of care and patient safety).

In the African context, family physicians have an important role in the primary or district hospital and can fill important skills gaps.^6^ Observational evidence associates family physicians with hospitals that have better performance and clinical processes and reduced risk of child mortality.^7^

Studies have not been able to detect any association between the supply of family physicians and routinely collected district health indicators.^8,9^ However, the number of family physicians is usually low and unlikely to have an impact that could be detected alongside many other confounding factors in this type of ecological evaluation. Better-performing districts may be more likely to deploy family physicians. There are no cost-effectiveness or economic evaluations of the contribution of family physicians, and no experimental studies on their effectiveness.

Clearly, we need more evidence to support advocacy, and a recent PRIMAFAMED e-workshop explored the types of measures that might be relevant. They suggested:

- Reduction in the number of referrals to higher levels of care.
- Improved quality and appropriateness of referrals to higher levels of care.
- More rational use of resources, such as investigations and prescribing of medication by clinical teams.
- Improved patient satisfaction and person-centredness.
- Improved comprehensiveness, such as surgical procedures.
- Improved quality of care, such as for non-communicable diseases.
- Reduction in adverse outcomes and possibly litigation.
- Improved and maintained competency and confidence in the clinical team.
- Increased research outputs from district health services.
- Strengthened palliative care.

What types of research studies would add value to the evidence base? In many countries, the number of family physicians is very small, making large-scale experimental or ecological studies impractical. In this context, research at the facility and district level is helpful. Qualitative studies can explore the perceptions of managers, healthcare workers and patients. A family physician impact assessment tool was developed in South Africa and could be adapted to other contexts, as the roles and job descriptions of family physicians may differ between countries. This allows a 360-degree evaluation of the family physician by co-workers.^10^ Case studies using mixed methods might also be helpful. These types of methods can generate evidence from countries with few family physicians.

In countries with larger numbers (e.g. South Africa, Nigeria, Kenya, and Tunisia) that are going to scale, researchers should be more ambitious. It may be helpful to monitor the placement and numbers of family physicians per district to enable observational studies with routinely collected health indicators. Cohort studies might be able to monitor change over time using more bespoke indicators. The Primary Care Assessment Tool measures the core functions and could be useful for comparative studies.^11^ It may also be possible to undertake more quasi-experimental or controlled studies, for example, using step wedge designs. Standardised process and patient outcome measures would be helpful for studies such as these.

My challenge to the family medicine community in Africa is to create this evidence and evaluate the value proposition of family physicians on our continent. The World Health Organization is looking for such evidence to ‘accelerate action on the global health and care workforce’ with a ‘special focus on the primary healthcare workforce in remote or otherwise vulnerable health settings’.^12^ Evidence is needed for advocacy at the regional level (e.g. World Health Organization Afro), at the national level (e.g. Departments of Health) and sub-national level (e.g. provinces, states, counties or even facilities).^13^ For the next 2 years, I am President of WONCA Africa and continue as Editor-in-Chief of the *African Journal of Primary Health Care & Family Medicine* (PHCFM) – I would like to challenge the readers to submit new evidence of the contribution of family physicians to health systems in Africa.
